# The impact of modern endourological techniques in the treatment of a century old disease - Medullary sponge kidney with associated nephrolithiasis

**Published:** 2013-12-25

**Authors:** P Geavlete, G Nita, E Alexandrescu, B Geavlete

**Affiliations:** “Sf. Ioan” Clinical Emergency Hospital, Bucharest, Romania

**Keywords:** endourological techniques, medullary sponge kidney, nephrolithiasis, renal disorders

## Abstract

Abstract

The remarkable progresses of imagistic and interventional techniques that have been implemented during the last decades facilitated the diagnostic and allowed the treatment indication changes for numerous renal disorders. The purpose of the present lecture was to outline a data review concerning a renal anomaly first described one century ago as well as to evaluate the impact of endourologic technical progresses over the therapeutic management of the respective disease. The medullary sponge kidney (MSK) or Cacchi-Ricci disorder represents a disturbance in the renal development characterized by the cystic type dilation and diffuse precalyceal ducts ectasias. The disease is also known as precalyceal tubular ectasia, pyramidal sponge kidney or cystic dilation of the renal collecting ducts MSK patients are most often asymptomatic, the diagnosis being emphasized in light of the investigations imposed by related complications such as renal stones, urinary tract infections (pyelonephritis), renal tubes acidosis or urine concentration defects.

The remarkable progresses of imagistic and interventional techniques that have been implemented during the last decades facilitated the diagnostic and allowed the treatment indication changes for numerous renal disorders. The purpose of the present lecture was to outline a data review concerning a renal anomaly first described one century ago as well as to evaluate the impact of endourologic technical progresses over the therapeutic management of the respective disease.

The medullary sponge kidney (MSK) or Cacchi-Ricci disorder represents a disturbance in the renal development characterized by the cystic type dilation and diffuse precalyceal ducts ectasias. More precisely, the modifications in question affect the intra-pyramidal or intrapapillary traject of medullary collecting ducts. These dilations are between 1 and 7 mm in size, frequently contain small calculi and the communication between the respective ducts and the calyces may present stenosis. On a microscopic level, a cubic, columnar and rarely transitional epithelium is described in the cystic areas, while isolated (closed) cysts display an atrophic epithelium [**1**].

The disease is also known as precalyceal tubular ectasia, pyramidal sponge kidney or cystic dilation of the renal collecting ducts [**2**]. Initially described by Guerrino Lenarduzzi in 1938 from the radiologic point of view, this disorder was afterwards correctly defined as a pathologic entity and correlated with the clinical features and radiologic changes by Roberto Cacchi si Vincenzo Ricci in 1948 [**3**].

It is a rare disease, characterized by a prevalence among the general population ranging between 5/10000 and 5/100000. This remote incidence is probably related to the fact that, in the majority of cases, the anomaly is asymptomatic and accidentally diagnosed in patients 30 to 50 years of age. Other authors sustain that MSK is established in 12-20% of the urolithiasis cases. Also, over 70% of the MSK patients develop renal calculi [**4**]. The disease is most frequently of bilateral nature and affects multiple renal pyramids, while the unilateral profile is rarely described [**5**]. The overall incidence as well as that of secondary lithiasis is higher in female patients.

MSK patients are most often asymptomatic, the diagnosis being emphasized in light of the investigations imposed by related complications such as renal stones, urinary tract infections (pyelonephritis), renal tubes acidosis or urine concentration defects. The disease is clinically visible in 0.2% up to 20% of cases, the most frequently encountered manifestations being represented by renal colic, micro- or macroscopic hematuria and fever [**4**].

The increased lithiasis incidence in MSK patients is not yet fully explained, as the majority of authors consider that the association of morphological anomalies (cystic dilations favoring the presence of urinary stasis) and functional disorders (hypercalciuria, hypocitraturia, distal renal tubes acidosis and defective acidification) create the conditions for stone formation (calcium phosphate and oxalate) [**6**]. The hyperparathyroidism is often present in MSK patients but recent data suggest that it does not intervene in the lithogenesis mechanism [**2**].

The MSK diagnostic relies exclusively on the imagistic methods. The kidney-ureter-bladder (KUB) simple X-ray emphasizes multiple calcifications located either uni- or bilaterally in the renal pyramids. Calculi may vary in size and radiologic intensity and are frequently gathered in groups. Renal ultrasound may display medullar hyperechogenicity due to the nephrolithiasis phenomenon.

Intravenous pyelography (IVP) constitutes the gold-standard MSK diagnostic modality. The main characteristics are represented by the presence of multiple calcifications and the contrast medium accumulation at the level of the dilated collecting ducts, thus creating the suggestive images of “bunches of grapes” or “bouquets of flowers” [**7**] (**Fig. 1**). Under the circumstances of extensive anomalies in all renal calyces, the kidney may be increased in size.

**Fig. 1:**
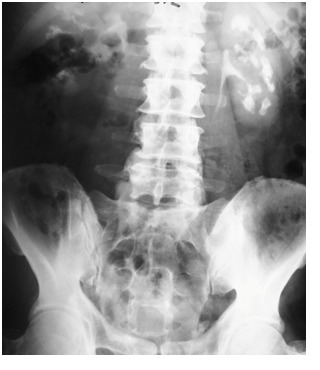
MSK patient with bilateral nephrocalcinosis on IVP

Standard computer tomography (CT) displays a lower sensitivity by comparison to IVP, emphasizing the associated lithiasis as well as the papillary calcifications but however lacking any specific aspects. Multidetector CT seems to be able to identify the particular MSK ducts’ modifications with a superior sensitivity when compared to IVP [**6**]. Magnetic resonance imaging (MRI) is rarely used in MSK diagnostic because of its remote sensitivity. 

 There is no specific treatment for MSK and the current therapeutic options are aimed at either avoiding complications or solving them. Also, no clinical practice guidelines have been published concerning the management, treatment or follow-up of MSK cases. In order to prevent complications in asymptomatic patients, many authors recommend regular urinary tests and KUB X-rays. In cases which are already symptomatic, the recommendation is to treat the associated metabolic imbalance and eventual nephrolithiasis using shock wave lithotripsy, ureteroscopy and percutaneous stone surgery [**1**].

 A retrospective analysis of all patients admitted in our clinical department between 2008 and 2013 identified 7 MSK cases with nephrocalcinosis. The diagnosis was radiologically established: cystic dilation of renal collecting ducts, nephrocalcinosis and renal or ureteral stones. 

 The symptoms of these 7 patients were represented by flank pain or renal colic, hematuria and urinary tract infection. Each patient had a normal renal function (serum creatinine between 0.8 and 1.5 mg/dlL at the time of the diagnosis). This parameter was not affected throughout the course of the hospital stay or subsequent follow-ups. One patient had a history of hyperparathyroidism, for which he received the proper treatment (parathyroidectomy).

 The 7 cases were treated as it follows: shock wave lithotripsy (1 patient) and flexible ureteroscopic holmium laser lithotripsy and pappillotomy (6 cases). The purpose of the endoscopic procedure was to clean the obstructed collecting ducts, which could have caused the respective symptoms (**Fig. 2**). The technique consists of vaporizing the urothelium found in the cystic dilations establishing a wider pathway, followed by lithotripsy (**Fig. 3-4**). 

**Fig. 2:**
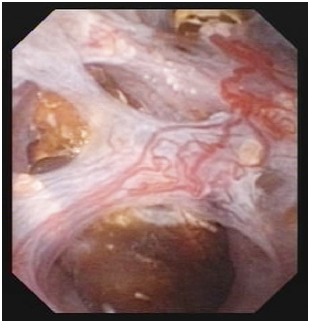
Ureteroscopic aspect of nephrocalcinosis

**Fig. 3:**
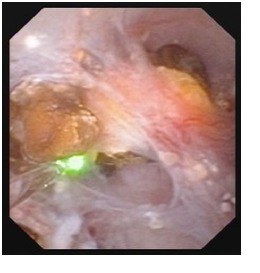
Urothelium vaporization in the cystic dilation

**Fig. 4:**
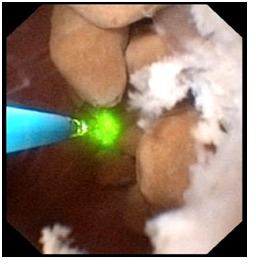
Holmium laser lithotripsy of multiple stones in a cystic dilatation

**Fig. 5:**
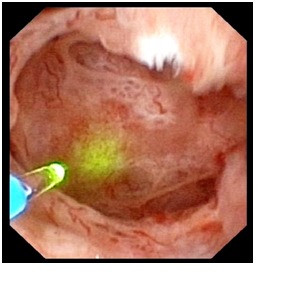
Clean collecting ducts at the end of the intervention

 The procedures were done under general anesthesia using digital Storz or Olympus ureteroscopes. A Dornier Medilas 20W laser, with 270 micron thick fibers was used for lithotripsy and papillotomy. For vaporization of the urothelium, the laser was set at 1000 - 1200 mJ, and for lithotripsy the set values were 500 - 700 mJ and 10 - 12 Hz. Ureteral access sheaths were used in every procedure.

 The procedure was considered successful when the lithotripsy process either turned the stones to dust or into fragments of under 3 mm. In all cases, a double J stent was placed at the end of the procedure.

 One of the patients required a single procedure for the technique to be successful, while in 5 of the other cases, multiple procedures were required (2 to 5) (**Fig. 6**). The most common stone composition was calcium oxalate monohydrate. The mean follow-up was 12 months and all patients remained asymptomatic. 

**Fig. 6:**
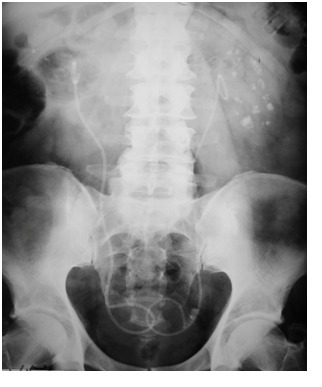
Post-operative KUB X-rays in a patient with bilateral MSK and nephrocalcinosis: 2 procedures on the right side and one on the left

The MSK prevalence in the general population is unknown and associated nephrolithiasis can be found in approximately 70% of cases. The underlying cause of stone disease in MSK patients is still not fully understood, while in symptomatic MSK cases of associated nephrolithiasis, ureteroscopic laser papillotomy is safe and effective. 
